# Structural Characterisation of FabG from *Yersinia pestis*, a Key Component of Bacterial Fatty Acid Synthesis

**DOI:** 10.1371/journal.pone.0141543

**Published:** 2015-11-05

**Authors:** Jeffrey D. Nanson, Jade K. Forwood

**Affiliations:** 1 School of Biomedical Sciences, Charles Sturt University, Wagga Wagga, NSW, 2678, Australia; 2 Graham Centre for Agricultural Innovation, Charles Sturt University, Wagga Wagga, NSW, 2678, Australia; University Paris Diderot-Paris 7, FRANCE

## Abstract

Ketoacyl-acyl carrier protein reductases (FabG) are ubiquitously expressed enzymes that catalyse the reduction of acyl carrier protein (ACP) linked thioesters within the bacterial type II fatty acid synthesis (FASII) pathway. The products of these enzymes, saturated and unsaturated fatty acids, are essential components of the bacterial cell envelope. The FASII reductase enoyl-ACP reductase (FabI) has been the focus of numerous drug discovery efforts, some of which have led to clinical trials, yet few studies have focused on FabG. Like FabI, FabG appears to be essential for survival in many bacteria, similarly indicating the potential of this enzyme as a drug target. FabG enzymes are members of the short-chain alcohol dehydrogenase/reductase (SDR) family, and like other SDRs, exhibit highly conserved secondary and tertiary structures, and contain a number of conserved sequence motifs. Here we describe the crystal structures of FabG from *Yersinia pestis* (*Yp*FabG), the causative agent of bubonic, pneumonic, and septicaemic plague, and three human pandemics. *Y*. *pestis* remains endemic in many parts of North America, South America, Southeast Asia, and Africa, and a threat to human health. *Yp*FabG shares a high degree of structural similarity with bacterial homologues, and the ketoreductase domain of the mammalian fatty acid synthase from both *Homo sapiens* and *Sus scrofa*. Structural characterisation of *Yp*FabG, and comparison with other bacterial FabGs and the mammalian fatty acid synthase, provides a strong platform for virtual screening of potential inhibitors, rational drug design, and the development of new antimicrobial agents to combat *Y*. *pestis* infections.

## Introduction

**Fig 1:**
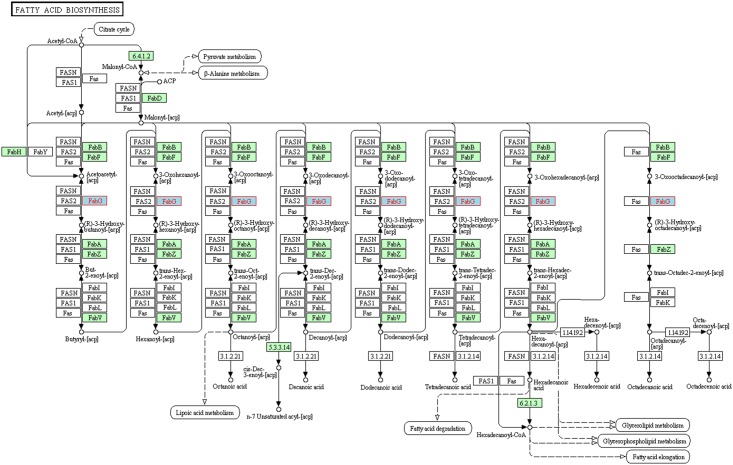
The fatty acid synthesis pathway of *Yersinia pestis*. In contrast to the mammalian fatty acid synthesis pathway in which reactions are catalysed by a single protein (FAS or FASN), each acyltransferase, condensation, reduction, and dehydration reaction of the fatty acid synthesis pathway of *Yersinia pestis* is catalysed by a discrete enzyme (highlighted green). FabG (highlighted blue with red lettering) is a highly conserved and ubiquitously expressed enzyme, which performs the first of two reduction reactions within the pathway. Homologues from other organisms are not highlighted. Image adapted from the KEGG PATHWAY database [14, 15].

Ketoacyl-acyl carrier protein reductases (FabG; EC 1.1.1.100) are highly conserved and ubiquitously expressed enzymes of the bacterial type II fatty acid synthesis (FASII) pathway, catalysing the reduction of the acyl carrier protein (ACP) linked β-ketoacyl molecules to β-hydroxyacyl-ACP thioesters necessary for the formation of saturated and unsaturated fatty acids. Such fatty acids are essential components of the many lipoproteins, phospholipids, and lipopolysaccharides that are incorporated into the bacterial cell envelope [1]. The FASII pathway is structurally distinct from the type I fatty acid synthesis (FASI) pathway of mammals and yeast, with the acyltransferase, condensation, reduction, and dehydration reactions of the pathway catalysed by discrete enzymes, in contrast to the multi-domain complex of the FASI pathway (FAS; also referred to by the gene name FASN) (Fig 1). In *Escherichia coli* and *Yersinia pestis*, the elongation of fatty acids typically begins with the condensation of malonyl-ACP and a fatty acyl-thioester catalysed by one of the three β-ketoacyl-ACP synthases (FabB, FabF, or FabH) forming a β-ketoacyl-ACP molecule, which is subsequently reduced by FabG. The product of this reaction is then dehydrated by a β-hydroxyacyl-ACP dehydrase (either FabA or FabZ) to yield enoyl-ACP. Another reductase of the FASII pathway, enoyl-ACP reductase (FabI), reduces enoyl-ACP, forming an acyl-ACP molecule. This acyl-ACP molecule can then re-enter the elongation cycle as a substrate for FabB or FabF, or be diverted to other pathways for the production of lipid molecules [1–3]. The structural differences between the FASI complex and the dissociated enzymes of the FASII pathway indicate the potential of FASII enzymes as antibacterial drug targets. Whilst the FASII enzyme enoyl-ACP reductase (FabI) has been the focus of numerous drug discovery efforts, some of which have led to clinical trials, few studies appear to be focused on FabG [4–9]. Like FabI, FabG appears to be essential for survival in many bacteria, similarly indicating the potential of this enzyme as a drug target [10–13].

FabG enzymes are members of the short-chain alcohol dehydrogenase/reductase (SDR) family (also known as the short chain oxidoreductase or SCOR family) [16]. SDR enzymes are known to catalyse a wide range of NAD(H) or NADP(H) dependent oxidoreduction reactions, and share a conserved nucleotide binding Rossmann fold motif consisting of a twisted β-sheet flanked by α-helices. SDR enzymes typically contain the NAD(P)(H) binding motif TGxxxGIG within the Rossmann fold, and catalytic tyrosine and lysine residues in a YxxxK sequence motif, as well as other fingerprint sequence motifs [17, 18]. The reaction mechanism of classical type SDRs, including FabG, is initiated by the transfer of a proton from the active site tyrosine and a hydride ion donated by the nicotinamide moiety of the co-factor. The active site lysine binds the co-factor and serves to lower the pKa of the tyrosine hydroxyl, facilitating the proton transfer and forming a proton relay with ordered solvent molecules to replenish the tyrosine proton donated during catalysis [19]. A conserved asparagine residue binds the active site lysine via an ordered water molecule, stabilizes the formation of this proton relay, and is essential for catalysis. Historically, a defining feature of the active sites of SDRs was the presence of this Ser-Tyr-Lys catalytic triad. It has been proposed that this active site triad be expanded to include the conserved asparagine residue, however others have argued the asparagine residue is not directly involved in catalysis and thus not part of a catalytic tetrad, leading to a discrepancy in the literature regarding the catalytic residues of SDR and FabG enzymes [19–21].

Here we describe the crystal structure of FabG from *Yersinia pestis* (*Yp*FabG). The causative agent of bubonic, pneumonic, and septicaemic plague, and three human pandemics, *Y*. *pestis* remains endemic in many parts of North America, South America, Southeast Asia, and Africa [22–25], and a threat to human health. Structural characterisation of *Yp*FabG may provide a platform for rational drug design and the development of new antimicrobial agents to combat *Y*. *pestis* infections.

## Materials and Methods

### Cloning, expression, and purification

The gene encoding *Y*. *pestis* FabG (GenBank accession number: AAM85326.1), a low molecular weight FabG of 244 amino acids, was cloned into the expression vector pMCSG21, solubly over-expressed, and purified as previously described [26]. Briefly, the gene encoding *Yp*FabG was amplified from genomic DNA and cloned into the expression vector pMCSG21 via ligation independent cloning. Plasmid containing recombinant *Yp*FabG was transformed into *E*. *coli* BL21(DE3) pLysS cells and expressed in auto-induction media [27] as a fusion protein containing a 6xHis tag and a Tobacco etch virus (TEV) protease cleavage site for tag removal. Cells were harvested by centrifugation, lysed by ~0.5 mg mL^-1^ lysozyme and two freeze/thaw cycles, and the cell lysate clarified by centrifugation. Soluble *Yp*FabG was purified by 6xHis affinity chromatography, and fractions containing *Yp*FabG were pooled, incubated with TEV protease for ~14 h at 4°C, and further purified by size-exclusion chromatography. Following size-exclusion chromatography, fractions containing *Yp*FabG were pooled, concentrated, and stored at -80°C.

### Crystallisation

Crystal screening was performed using the hanging-drop vapour-diffusion technique and commercial screens. Initial diffraction-quality crystals were obtained in 300mM ammonium phosphate monobasic, 30% (w/v) glycerol, using a protein concentration of 35mg ml^-1^ as previously reported [26]. The electron density maps generated from the previously reported crystallization data indicated a high degree of disorder in half of the asymmetric unit (ASU). As such, crystallisation conditions were further optimised using an additive screen (Hampton Research), with droplets consisting of 1.5μl of protein solution, and 1.5μl of a 1:1 mix of additive and reservoir solutions, mixed and suspended over 300 μL of reservoir solution (300mM ammonium phosphate monobasic). After one week, two conditions formed crystals of sufficient quality for data collection; one obtained with 300mM ammonium phosphate monobasic and 30% (w/v) trimethylamine N-oxide dihydrate (TMAO) solution, and the other with 300mM ammonium phosphate monobasic and 100 mM sodium bromide (NaBr).

### Data collection, structure determination, refinement, and analysis

Prior to data collection, all *Yp*FabG crystals were cryoprotected in 35% glycerol and flash-cooled in liquid nitrogen. Diffraction data were collected on the MX1 beamline at the Australian synchrotron. A total of 200° of data with 0.5° oscillations were collected at a wavelength of 0.7108 Å for both crystals. Diffraction data were indexed, integrated, and scaled using iMosflm [28] and Aimless [29] from the CCP4 suite [30, 31]. Diffraction data for the crystal obtained in the presence of TMAO were scaled to 2.50 Å, and diffraction data for the crystal obtained in the presence of NaBr were scaled to 2.85 Å. The *Yp*FabG crystals displayed different space groups, with the TMAO crystal displaying P4_2_2_1_2 symmetry, with the unit cell lengths *a* = 88.21, *b* = 88.21, *c* = 54.21 Å, and the NaBr crystal displaying P12_1_1 symmetry, with the unit cell parameters *a* = 64.74, *b* = 96.85, *c* = 71.55 Å, α = 90, β = 104.91, γ = 90°. The structures were solved by molecular replacement performed with Phaser [32], using a monomer of *Yp*FabG built from previously acquired data [26] as the search model. Further model building and refinement was performed using Coot [33] and PHENIX [34]. Structural renderings were performed in PyMol. The quaternary structure of *Yp*FabG and protein interfaces were investigated using the Protein Interfaces, Surfaces, and Assemblies’ service (PISA) from the European Bioinformatics Institute (http://www.ebi.ac.uk/pdbe/prot_int/pistart.html) [35]. Sequence alignments were generated using the T-Coffee (http://tcoffee.crg.cat/) [36, 37] and ESPript (http://espript.ibcp.fr/) [38] web services. Structural alignments were performed using the Protein structure comparison service (PDBeFold) at the European Bioinformatics Institute (http://www.ebi.ac.uk/msd-srv/ssm) [39, 40]. A summary of the crystallographic and refinement statistics for *Yp*FabG structures are provided in Table 1.

**Table 1:** Data processing and model statistics.

	*Yp*FabG TMAO	*Yp*FabG NaBr
PDB ID	5CEJ	5CDY
Resolution range (Å)	31.17–2.50 (2.60–2.50)	30.15–2.85 (3.00–2.85)
Space group	P4_2_2_1_2	P12_1_1
Unit cell length (Å)	*a* = 88.21, *b* = 88.21, *c* = 54.21	*a* = 64.74, *b* = 96.85, *c* = 71.55
Unit cell angle (°)	α = 90, β = 90, γ = 120	α = 90, β = 104.91, γ = 90
Total observations	114120 (12891)	78320 (11225)
Unique reflections	7814 (854)	19603 (2831)
Multiplicity	14.6 (15.1)	4.0 (4.0)
Completeness (%)	99.9 (100)	98.1 (98.0)
Mean I/sigma (I)	9.7 (3.0)	9.4 (2.0)
Mean CC (1/2)	0.992 (0.908)	0.994 (0.555)
R-pim	0.055 (0.228)	0.093 (0.545)
R-meas	0.213 (0.897)	0.136 (0.808)
R-merge	0.205 (0.867)	0.100 (0.594)
R-work	0.2198	0.2136
R-free	0.2550	0.2479
Number of atoms*	1713	6157
RMSD bonds (Å)	0.004	0.003
RMSD angles (°)	0.849	0.649
Ramachandran favoured (%)	95.7	96.2
Ramachandran allowed (%)	4.3	3.8
Ramachandran outliers (%)	0	0

Values in parentheses are for highest resolution shell.

* Not including hydrogen atoms.

### Enzyme assays

The reductase activity of *Yp*FabG was measured by the decrease of absorbance at 340 nm as a function of NADH or NADPH oxidation using a molar extinction coefficient of 6220 M^-1^ cm^-1^, similarly to that described previously [7, 9, 13, 41, 42]. Reaction mixtures contained co-factor, acetoacetyl-CoA, and 0.10 μg of recombinant *Yp*FabG, made to a final volume of 100 μL in a 96 well UV-transmissible plate with a solution of 125 mM NaCl and 25 mM HEPES pH 7.4, with the reaction initiated by the addition of substrate. NADH/NADPH oxidation without the addition of acetoacetyl-CoA served as a blank. Each assay was performed in at least triplicate. The decrease in absorbance at 340 nm was recorded over 4 min, and readings for the linear portion of the assay (typically over 45 s) were used in analyses. Co-factor specificity was determined using 200 μM co-factor and 500 μM acetoacetyl-CoA. K_m_ and V_max_ were determined by varying the concentration of either NADPH or acetoacetyl-CoA, while maintaining a fixed concentration of the other. K_m_ and V_max_ were calculated via nonlinear regression analyses with GraphPad Prism.

## Results and Discussion

### Overall structure of *Yp*FabG

To determine the high resolution crystal structure of FabG from *Y*. *pestis* (*Yp*FabG), a potentially important drug target, recombinant *Yp*FabG was expressed and purified from *E*. *coli*, and crystallised by hanging drop vapour diffusion. Recombinant *Yp*FabG crystallised in two different crystal forms; *Yp*FabG crystallised in the presence of TMAO (*Yp*FabG TMAO) diffracted to 2.5 Å and contained one monomer in the ASU, with electron density visible for 234 of 244 residues (residues 2–189, and 197–241), and was refined to Rwork/Rfree values of 0.220/0.255. *Yp*FabG crystallised in the presence of NaBr (*Yp*FabG NaBr) diffracted to 2.85 Å and contained a homotetramer in the ASU, with electron density visible for 832 of 976 residues, with disordered regions between residues 139–147 and 185–206 in each monomer, and was refined to Rwork/Rfree values of 0.214/0.248. Overall, the structures were similar, with an RMSD of 0.35 Å over 205 amino acids, however they also contained some important structural differences.

**Fig 2:**
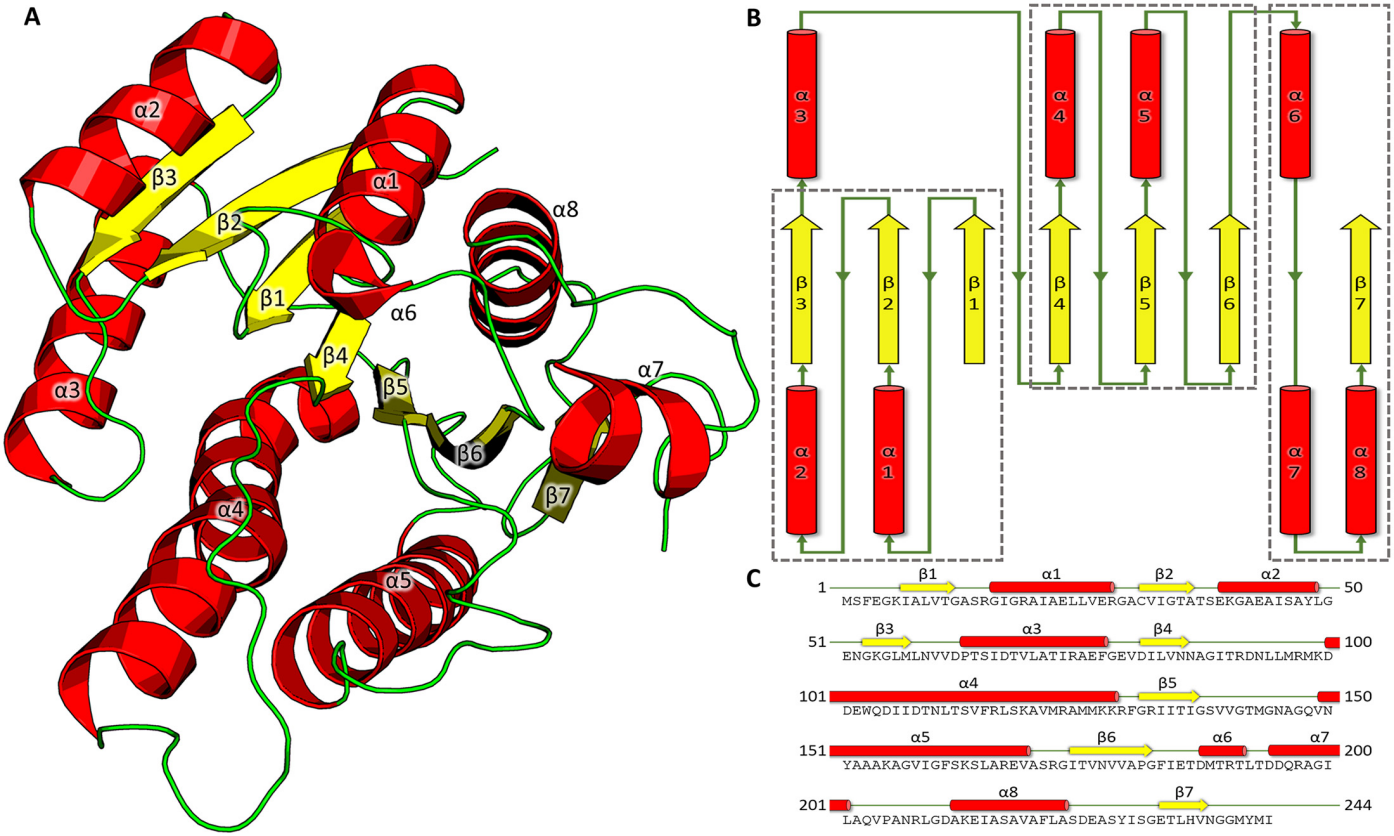
The tertiary structure of *Yersinia pestis* FabG (*Yp*FabG). **(A)** A cartoon representation of *Yp*FabG TMAO showing the central seven strand twisted β-sheet flanked either side by α-helices. **(B)** A 2D depiction of the secondary structure features of *Yp*FabG, with the two βαβαβ motifs and αααβ motif highlighted by grey squares. **(C)** The amino acid sequence of *Yp*FabG, and corresponding secondary structure features. β-sheets are displayed yellow; α-helices are displayed red; loop regions are displayed green.

The refined structures of the two crystal forms of *Yp*FabG reveal that the protein contains a typical Rossmann fold motif, comprised of a seven strand twisted β-sheet surrounded by eight α-helices. *Yp*FabG can be divided into three motifs, two βαβαβ motifs and a single αααβ motif (Fig 2). The first βαβαβ motif is comprised of β-strands β1, β2, and β3, and α-helices α1 and α2, and is connected to the second βαβαβ motif, containing β-strands β4, β5, and β6, and α-helices α4 and α5, by helix α3. The final motif contains a helix-turn-helix feature formed by α6 and α7 that lies away from the central β-sheet of the Rossmann fold, and helix α8 and strand β7, which align adjacent to strand β6. Overall, these secondary structure elements are arranged in a topology of β1-α1-β2-α2-β3—α3—β4-α4-β5-α5-β6—α6-α7-α8-β8 (Fig 2). These secondary structure elements are clearly observable in the *Yp*FabG TMAO structure, with only residues 190–197, which form part of the helix-turn-helix motif comprised of helices α6 and α7, and the loop region connecting the two helices, unmodelled due to disorder. Conversely, this helix-turn-helix motif is completely disordered in our *Yp*FabG NaBr structure.

**Fig 3:**
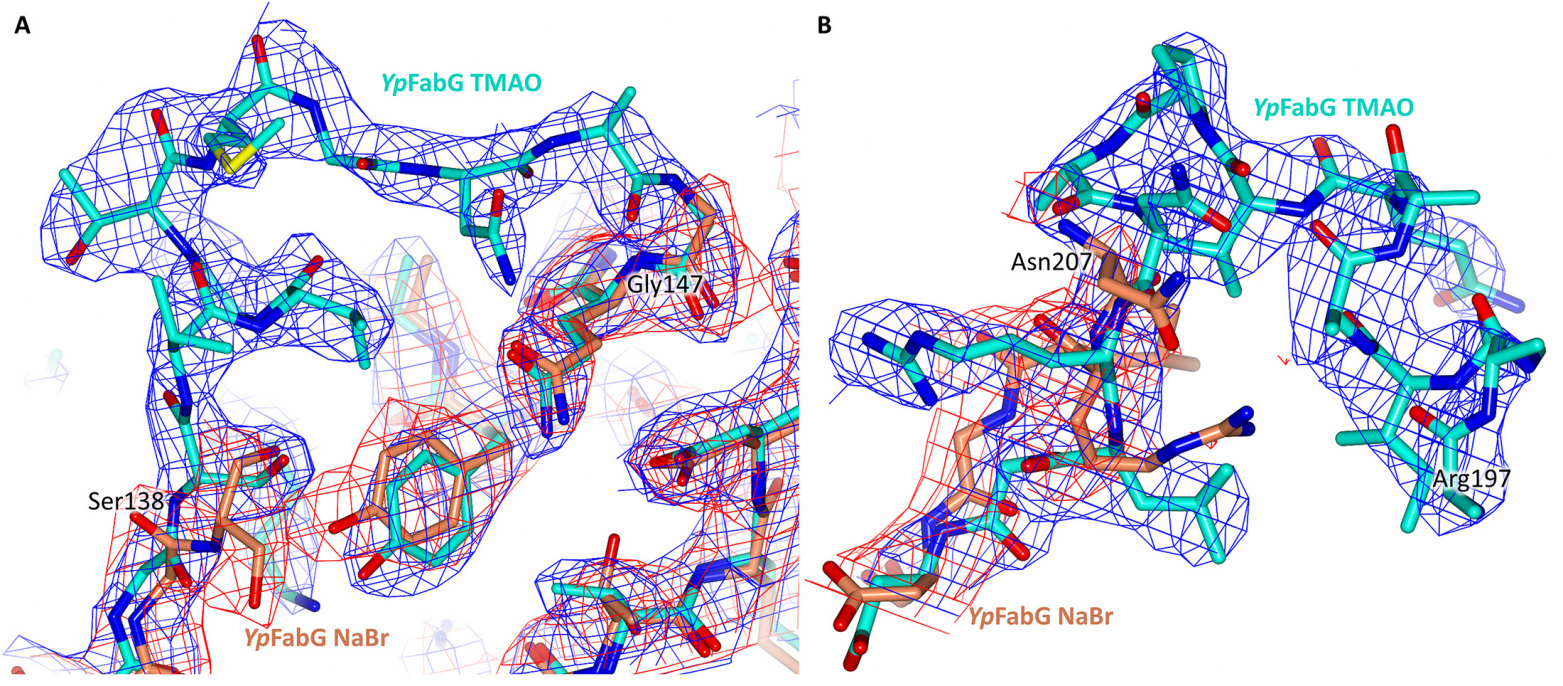
Different *Yersinia pestis* FabG (*Yp*FabG) crystal forms exhibit different loop conformations. Despite similar crystallisation conditions, the structures of *Yp*FabG TMAO and *Yp*FabG NaBr exhibit differences within the loop regions surrounding the co-factor binding pocket. **(A)** Within our *Yp*FabG NaBr structure (orange model), the region between Ser138 and Gly147 is highly disordered, with little visible electron density (red mesh), however this region is ordered in our *Yp*FabG TMAO structure (cyan model; blue electron density map). **(B)** The helix-turn-helix motif of both *Yp*FabG structures exhibit some disorder, however a greater portion of this motif is visible in the *Yp*FabG TMAO structure. Electron density maps are 2Fo-Fc maps contoured to 1σ.

Despite similar crystallisation conditions, several differences were observed between the structures of *Yp*FabG TMAO and *Yp*FabG NaBr, including large differences surrounding the co-factor binding pocket. The conformation of the helix-turn-helix motif found in the *Yp*FabG TMAO structure closely resembles that of *E*. *coli* FabG bound to NADP^+^ (PBD entry 1q7b; [19]), despite the apparent absence of bound co-factor in our structure. In contrast, a greater portion of the helix-turn-helix motif is disordered in the *Yp*FabG NaBr structure, with little visible electron density in this region (Fig 3). The ordered residues of this motif appear to adopt a conformation similar to that of unbound *E*. *coli* FabG (PBD entry 1i01; [16]), with superposition of both the NADP^+^ bound and unbound *E*. *coli* structures onto the *YpFabG* structures revealing the helix-turn-helix motif of *Yp*FabG NaBr is incompatible with NADPH binding, indicating this region is highly flexible. Furthermore, the loop joining β-strand β5 and α-helix α5 within the *Yp*FabG NaBr structure is largely disordered, with superposition of NADP^+^ bound and unbound *E*. *coli* FabG structures indicating that Ser138 located at the start of this loop region must move ~3 Å to accommodate the nicotinamide portion of NADP^+^. In comparison, this region is ordered within the *Yp*FabG TMAO structure, and adopts a conformation almost identical to that of *E*. *coli* FabG in complex with NADP^+^ [19]. The cause of this conformational change is not apparent. The conformation that appears to mimic that of *E*. *coli* FabG in complex with NADP^+^ could possibly be induced by crystal contacts, or the presence of TMAO within a binding pocket that becomes occupied by the 2’-phosphate of NADPH upon co-factor binding [19]. A similar conformation was observed in the crystal structure of *Plasmodium falciparum* FabG [7], where a sulphate ion bound within the same phosphate binding pocket of the NADPH binding site appeared to induce a conformation mimicking that of the NADPH bound active conformation previously observed in *E*. *coli* FabG [7, 19]. A single phosphate molecule is observed within the same location in our *Yp*FabG NaBr structure, however the presence of this molecule in only one monomer may not be sufficient to induce this active conformation.

The conformational changes and flexibility observed in the NADP(H) binding site of *Yp*FabG and *E*. *coli* FabG may pose an issue for structure-based drug design or virtual screening of potential antimicrobial agents, thus such studies should consider that inhibitors which demonstrate competitive binding in regards to NADPH, or mimic NADPH, may be incompatible without first taking into account conformation changes within this binding site.

### Co-factor specificity of *Yp*FabG

**Fig 4:**
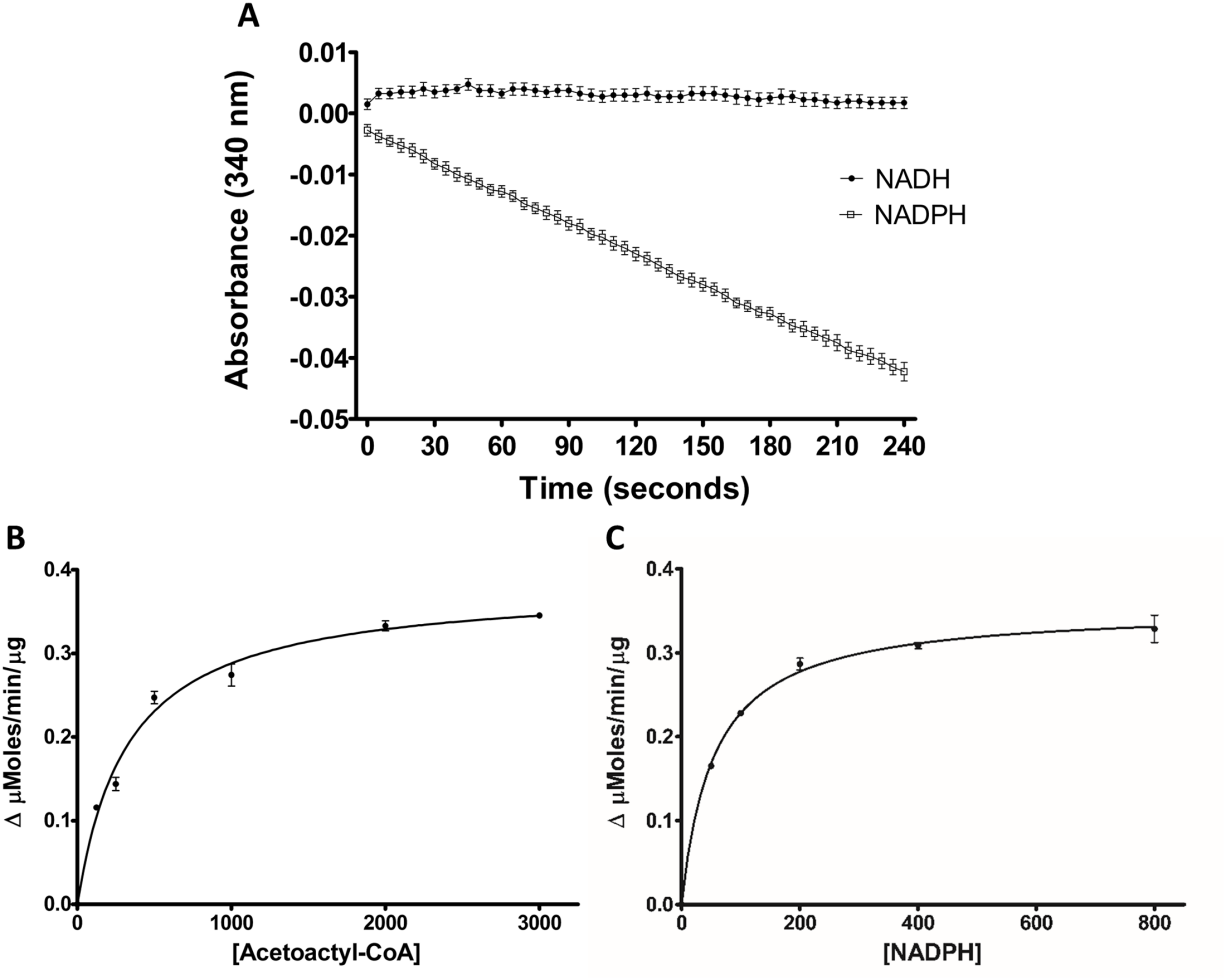
The co-factor specificity and enzymatic activity of *Yersinia pestis* FabG (*Yp*FabG). **(A)** The activity of *Yp*FabG in the presence of NADH (•) or NADPH (□) confirmed *Yp*FabG reductase activity is highly dependent upon NADPH as the co-factor. *Yp*FabG was assayed in the presence of acetoacetyl-CoA and NADPH, by varying the concentration of either acetoacetyl-CoA **(B)** or NADPH **(C)** while maintaining a fixed concentration of the other. The K_m_ values of acetoacetyl-CoA and NADPH were 329.9 ± 31.2 μM and 55.1 ± 3.8 μM respectively.

The structures of *Yp*FabG, particularly the presence of a charged pocket for the 2’ phosphate of NADPH, indicated the catalytic mechanism of *Yp*FabG to be NADPH dependent. To confirm this, the ketoacyl reductase activity of *Yp*FabG was assessed by the oxidation of NADH or NADPH in the presence of acetoacetyl-CoA, similarly to that described previously [7, 9, 13, 41, 42]. The enzymatic activity assays confirmed *Yp*FabG reductase activity is highly dependent upon NADPH as the co-factor, with little reductase activity occurring in the presence of NADH (Fig 4). *Yp*FabG was found to catalyse the reduction of acetoacetyl-CoA with a K_m_ of 329.9 ± 31.2 μM in the presence of 0.9 mM NADPH. The K_m_ for NADPH was 55.1 ± 3.8 μM in the presence of 1.5 mM acetoacetyl-CoA. V_max_ with respect to acetoacetyl-CoA was 0.383 ± 0.010 μmoles min^-1^ μg^-1^.

### Quaternary structure of *Yp*FabG

Despite very similar crystallisation conditions, the *Yp*FabG TMAO crystal displays P4_2_2_1_2 symmetry with a single *Yp*FabG monomer within the ASU, whereas the *Yp*FabG NaBr crystal displays P12_1_1 symmetry with four *Yp*FabG monomers within the ASU arranged to form a homotetramer. Crystallographic symmetry of *Yp*FabG TMAO arrange to form a tetramer highly similar to that within the ASU of *Yp*FabG NaBr, with superposition of the *Yp*FabG NaBr structure and the tetramer of *Yp*FabG TMAO generated through crystallographic symmetry indicating no clashes that would alter crystal packing. The only obvious difference between the two tetramers is the presence of the loop joining β-strand β5 and α-helix α5 (residues 139–147), and the helix-turn-helix motif (residues 185–205) in the *Yp*FabG TMAO structure, which are largely disordered in the *Yp*FabG NaBr structure (Fig 3). These regions appear to alter crystal packing, with symmetry generated *Yp*FabG TMAO tetramers packing in parallel, and the helix-turn-helix motifs of each monomer packing against the same motif of the symmetry mate, whilst symmetry mates of the *Yp*FabG NaBr structure appear to form crystal contacts primarily through helices α1, α2, and α3 at the corner of each tetramer.

**Fig 5:**
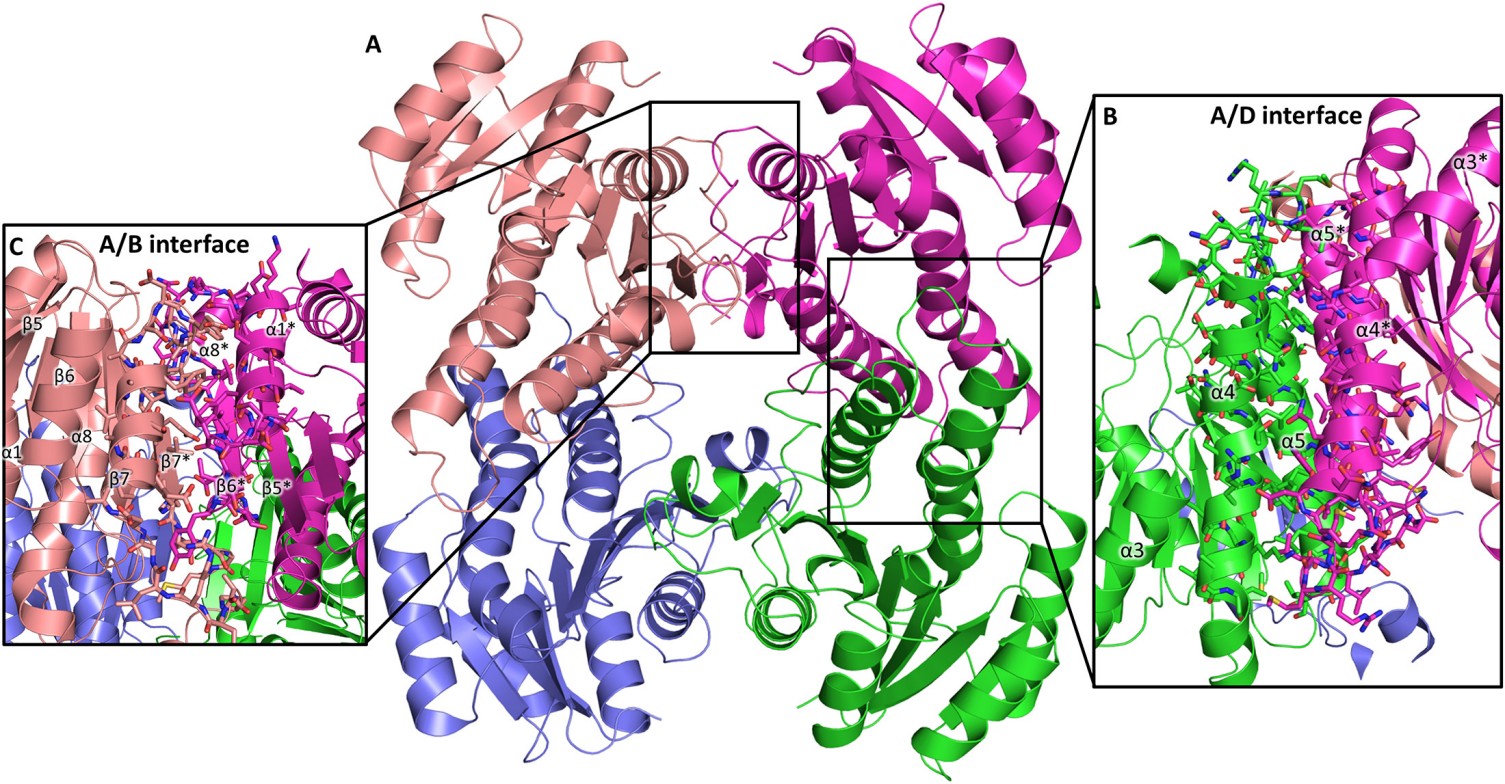
The quaternary structure of *Yersinia pestis* FabG (*Yp*FabG). **(A)** A cartoon representation of the *Yp*FabG TMAO homo-tetramer, the biological unit as indicated by PISA. **(B)** *Yp*FabG forms a tetramer with two types of interfaces. The larger of the two interfaces in terms of buried surface area (A/D interface) is formed primarily through interactions between helices α4 and α5 of each monomer, which align antiparallel to form a four helix bundle. **(C)** The smaller of the two interfaces (A/B interface) is formed through interactions between helix α8, strand β7, and the adjoining loop region of each monomer, which align antiparallel, with the β7 strand of each monomer arranged to form a contiguous 14 strand β-sheet that spans the dimer interface. Opposing monomer secondary structure features are indicated by asterisks (*).

Analysis of the quaternary structure of *Yp*FabG TMAO using PISA predicts the biological unit of *Yp*FabG to be a tetramer, with symmetry similar to that observed in the *Yp*FabG NaBr ASU. The *Yp*FabG tetramer is formed through two types of interfaces (Fig 5). The largest interface in terms of buried surface area is between chains A and D, and B and C (A/D interface), comprising 1,653 Å^2^ (~15%) of the total surface area with an estimated binding energy of -119 kJ mol^-1^, formed through interactions between helices α4 and α5 of each monomer, which align antiparallel to form a four helix bundle (Fig 5). Whilst the interface between helices α4 and α5 and counterparts on the opposing monomer appear to be formed primarily through hydrophobic packing, PISA analysis suggests the interface is also stabilised by several hydrogen bonds and four salt bridges (Table 2). The smaller of the two interfaces exists between chains A and B, and C and D (A/B interface), comprising 1360 Å^2^ (~12%) of the total surface area, with an estimated binding energy of -97 kJ mol^-1^. In contrast to the larger interface between helices α4 and α5 of each monomer, this interface appears to be predominantly formed by hydrogen bonds and salt bridges (Table 2). These interactions are mostly between helix α8, strand β7, and the adjoining loop region of each monomer, which align in an antiparallel fashion, with the β7 strand of each monomer arranged to form a contiguous 14 strand β-sheet that spans the dimer interface (Fig 5).

**Table 2:** Hydrogen bonds and salt bridges of the *Yp*FabG dimer interfaces.

**Interactions within the A/D interface**					
**Hydrogen bonds**			**Salt bridges**		
**Chain A**	**Dist. [Å]**	**Chain D**	**Chain A**	**Dist. [Å]**	**Chain D**
LYS 119 [NZ]	3.61	LEU 95 [O]	ARG 116 [NH2]	3.14	ASP 100 [OD2]
LYS 119 [NZ]	2.73	MET 98 [O]	ARG 116 [NE]	3.09	ASP 100 [OD2]
ARG 116 [NH2]	3.14	ASP 100 [OD2]	ASP 100 [OD2]	3.09	ARG 116 [NE]
ARG 116 [NE]	3.09	ASP 100 [OD2]	ASP 100 [OD2]	3.14	ARG 116 [NH2]
TRP 103 [NE1]	3.11	THR 112 [OG1]			
LYS 163 [NZ]	2.51	THR 142 [O]			
ARG 167 [NH2]	2.80	MET 143 [O]			
SER 164 [OG]	2.21	ASN 145 [O]			
ASN 145 [N]	3.90	SER 164 [OG]			
GLY 147 [N]	2.96	GLU 168 [OE1]			
LEU 95 [N]	2.50	GLU 168 [OE2]			
LEU 95 [O]	3.61	LYS 119 [NZ]			
MET 98 [O]	2.73	LYS 119 [NZ]			
ASP 100 [OD2]	3.09	ARG 116 [NE]			
ASP 100 [OD2]	3.14	ARG 116 [NH2]			
THR 112 [OG1]	3.11	TRP 103 [NE1]			
THR 142 [O]	2.51	LYS 163 [NZ]			
MET 143 [O]	2.80	ARG 167 [NH2]			
ASN 145 [O]	2.21	SER 164 [OG]			
SER 164 [OG]	3.90	ASN 145 [N]			
GLU 168 [OE1]	2.96	GLY 147 [N]			
GLU 168 [OE2]	2.50	LEU 95 [N]			
**Interactions within the A/B interface**					
**Hydrogen bonds**			**Salt bridges**		
**Chain A**	**Dist. [Å]**	**Chain B**	**Chain A**	**Dist. [Å]**	**Chain B**
SER 171 [N]	3.67	PRO 205 [O]	ARG 28 [NH2]	3.41	GLU 226 [OE1]
TYR 229 [OH]	2.99	ARG 208 [O]	ARG 28 [NH1]	3.61	GLU 226 [OE2]
TYR 229 [N]	3.78	GLU 214 [OE1]	ARG 28 [NH2]	2.80	GLU 226 [OE2]
ARG 28 [NH2]	2.80	GLU 226 [OE2]	ARG 167[NH1]	3.72	TYR 242 [O]
SER 217 [OG]	3.17	GLU 226 [OE2]	ARG 167[NH2]	2.52	TYR 242 [O]
ARG 208 [NH2]	3.50	SER 228 [OG]	GLU 226 [OE1]	3.41	ARG 28 [NH2]
ASN 238 [N]	3.28	TYR 229 [O]	GLU 226 [OE2]	3.61	ARG 28 [NH1]
GLY 239 [N]	2.62	TYR 229 [O]	GLU 226 [OE2]	2.80	ARG 28 [NH2]
HIS 236 [N]	2.86	GLU 233 [OE2]	TYR 242 [O]	3.72	ARG 167[NH1]
ARG 167 [NH2]	2.52	TYR 242 [O]	TYR 242 [O]	2.52	ARG 167[NH2]
PRO 205 [O]	3.67	SER 171 [N]			
ARG 208 [O]	2.99	TYR 229 [OH]			
GLU 214 [OE1]	3.78	TYR 229 [N]			
GLU 226 [OE2]	2.80	ARG 28 [NH2]			
GLU 226 [OE2]	3.17	SER 217 [OG]			
SER 228 [OG]	3.50	ARG 208 [NH2]			
TYR 229 [O]	3.28	ASN 238 [N]			
TYR 229 [O]	2.62	GLY 239 [N]			
GLU 233 [OE2]	2.86	HIS 236 [N]			
TYR 242 [O]	2.52	ARG 167 [NH2]			

### Comparison of *Yp*FabG with the ketoreductase domain of mammalian FAS and SDRs

**Fig 6:**
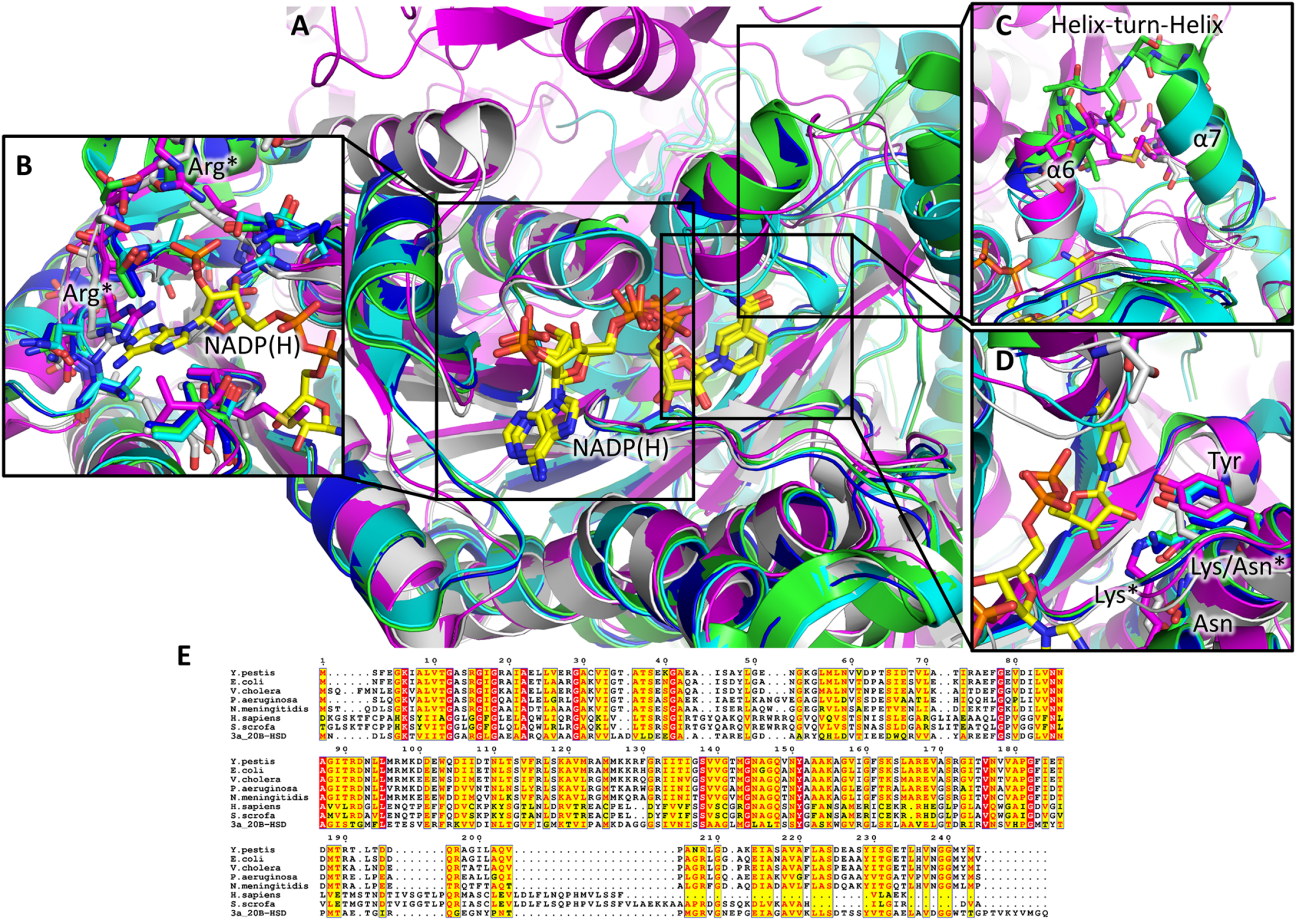
Comparison of bacterial FabG enzymes and the ketoreductase domain of the mammalian FAS. **(A)** Superposition of FabG from *Y*. *pestis* (*Yp*FabG TMAO; blue), *E*. *coli* (green), *V*. *cholerae* (cyan), and the ketoreductase (KR) domain of the mammalian FAS from *S*. *scrofa* (magenta) and *H*. *sapiens* (silver) reveals a highly conserved tertiary structure, however there are several differences surrounding the NADP(H) (yellow) binding site. **(B)** The mammalian FAS enzyme possesses two additional electropositive arginine residues, which appear to interact with the 3’ ribose phosphate of NADP(H) compared to bacterial FabG enzymes. **(C)** Additionally, the helix turn helix motif that caps the co-factor binding site appears shorter in the mammalian FAS compared to bacterial FabG enzymes. **(D)** Interestingly, the position of the active site lysine and the structurally conserved asparagine in the active site of the KR domain of the mammalian FAS appears switched in comparison to bacterial FabG enzymes, yet the position of the active site tyrosine is unchanged. Residues of *H*. *sapiens* and *S*. *scrofa* FAS are indicated by asterisks (*); active site residues common to both *Yp*FabG and the mammalian FAS are not marked. **(E)** Sequence alignment of FabG from *E*. *coli*, *V*. *cholerae*, *N*. *meningitidis*, *P*. *aeruginosa*, 3α, 20β-hydroxysteroid dehydrogenase, and the KR domain of the mammalian FAS from *H*. *sapiens* and *S*. *scrofa*. Conserved residues are highlighted yellow; strictly conserved residues are highlighted red.

A structural homology search of the PDB database using PDBeFold shows the most similar enzymes to *Yp*FabG are FabG enzymes from *E*. *coli* (PBD entry 1q7b; [19]), *Vibrio cholera* (PBD entry 3rsh; unpublished), *Pseudomonas aeruginosa* (PBD entry 3rsh; [8]) and *Neisseria meningitidis* (PBD entry 4m8s; unpublished), with RMSD values of 0.59 Å over 234 residues, 0.67 Å over 229 residues, 0.85 Å over 210 residues, and 1.01 Å over 228 residues, respectively. *Yp*FabG also shares a high degree of structural homology with the ketoreductase (KR) domain of the mammalian FAS enzymes from both *Sus scrofa* (PDB entry 2vz8; [43]) and *Homo sapiens* (PDB entry 4PIV; [44]) (Fig 6), with RMSD values of 2.24 Å over 208 amino acids and 2.10 Å over 205 amino acids, respectively, and with 3α, 20β-hydroxysteroid dehydrogenase (PDB entry 2hsd; [45]), the first member of the SDR family for which the crystal structure was reported, with an RMSD of 1.34 Å over 230 amino acids, despite sequence identities of 18%, 19% and 26% respectively. Furthermore, FabG exhibits little deviation from the 40 highly conserved fingerprint motifs observed in 3α, 20β-hydroxysteroid dehydrogenase, and characteristic of TGxxxGIG SDRs [17, 18]. Only 1 out of 40 residues (Asn59Asp) deviates from the highly conserved fingerprint residues, with this alteration also present in *E*. *coli*, *V*. *cholerae*, and *N*. *meningitidis* FabG homologues.

**Fig 7:**
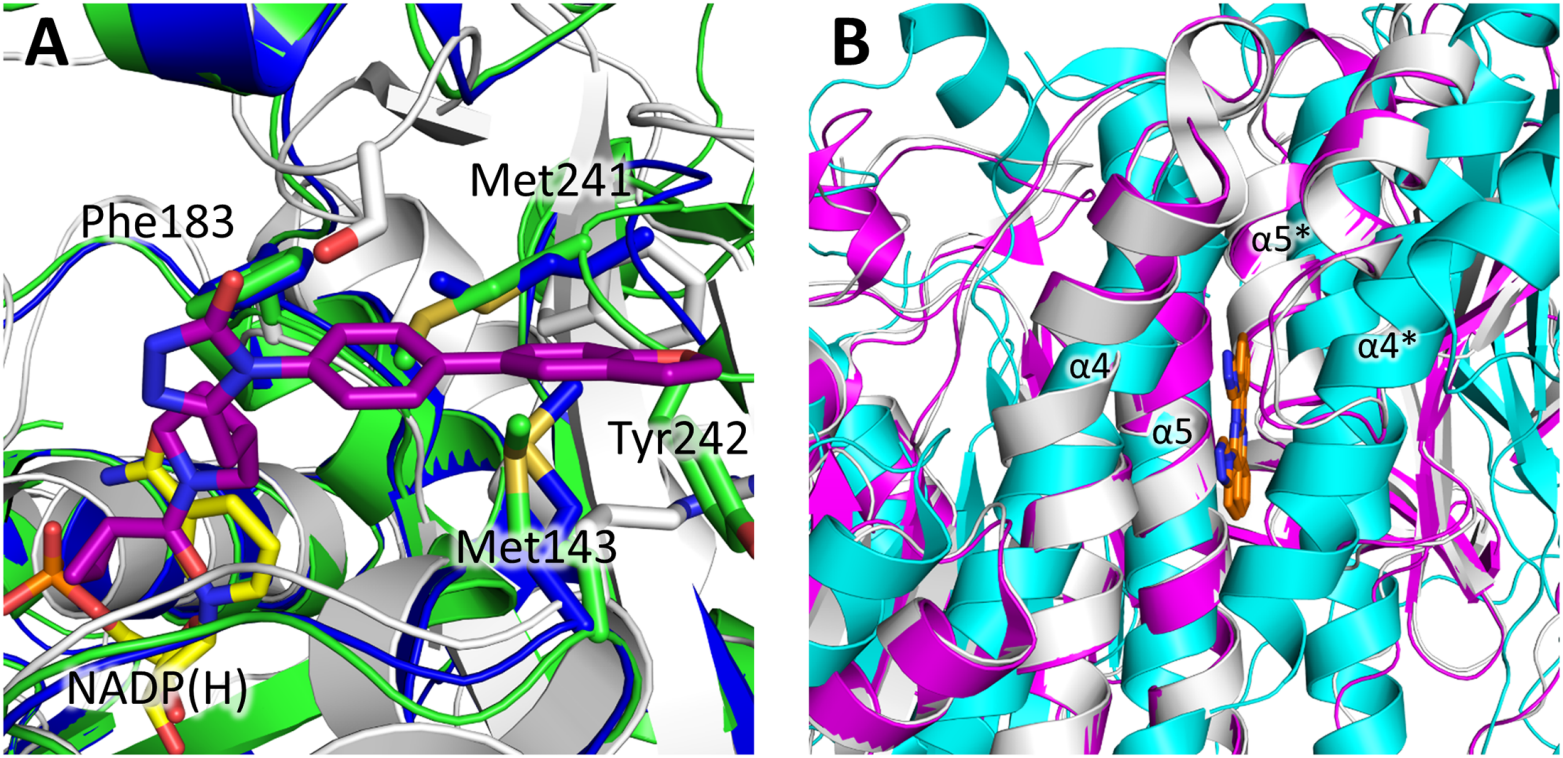
Comparison of bacterial FabG and mammalian FAS ketoreductase domain inhibitor binding sites. **(A)** The mammalian FAS ketoreductase (KR) domain (silver) inhibitor GSK2194069 (purple) binding site extends from the KR domain active site into the interface between the KR and pseudo-methyltransferase domains of the FAS complex [44]. In *Yp*FabG (blue) and *E*. *coli* FabG (green) this site extends towards the A/B dimer interface at the centre of the tetramer, and appears incompatible with GSK2194069 binding due to clashes with residues Met143, Phe183, Met241, and Tyr242. **(B)** The allosteric inhibitors (orange) reported by Cukier et al. (2013) bind within the A/D dimer interfaces of FabG between helices α4 and α5 of each monomer, distorting the NADPH binding pocket to prevent co-factor binding [8]. Whilst the KR domain of mammalian FAS does not possess the same interfaces, superposition of bacterial FabG (cyan) and the mammalian FAS KR domains of *H*. *sapiens* (silver) and *S*. *scrofa* (magenta) reveals a region somewhat homologous to the A/D interface. However, two of the four helices of this site are truncated in comparison to FabG, thus such structural differences may be able to be exploited to reduce the affinity of the inhibitor for the mammalian FAS. Opposing monomer secondary structure features are indicated by asterisks (*).

Despite this highly conserved tertiary structure, there are significant structural differences between FabG enzymes and the KR domain of the mammalian FAS surrounding the co-factor binding site (Fig 6), including a rearrangement of the active site lysine and the structurally conserved asparagine required for catalysis in the mammalian FAS, and configuration of the electropositive arginine residues surrounding the 3’ phosphate of NADP(H). The helix turn helix motif that caps the co-factor binding site also appears shorter in both the human and *S*. *scrofa* mammalian FAS compared to bacterial FabG enzymes (Fig 6), however this could be due to the inherent flexibility of this region. Comparison between bacterial FabG enzymes and the structure of the human FAS KR domain bound by the inhibitor GSK2194069 (PDB entry 4PIV; [44]) highlights important differences within the quaternary structure of the mammalian FAS and that of bacterial FabG enzymes. GSK2194069 binds the KR domain of human FAS along a groove that extends from the KR domain active site, adjacent to the nicotinamide portion of the co-factor, into the interface between the KR domain and the pseudo-methyltransferase domain of the FAS complex [44]. Within the *Yp*FabG and *E*. *coli* FabG structures, this groove extends towards the A/B dimer interface and the center of the tetramer, with superposition of the KR domain in complex with GSK2194069, *Yp*FabG, and *E*. *coli* FabG structures indicating GSK2194069 is incompatible with these bacterial FabG enzymes, clashing with residues Met143, Phe183, Met241, and Tyr242, which protrude into this groove (Fig 7A).

Such differences may be exploited during the design of new antimicrobials targeting the bacterial FabG enzymes, potentially reducing their affinity for the mammalian FAS complex and thus limiting adverse host effects. For example, inhibitors that rely upon interactions with Met143, Phe183, Met241, and Tyr242, or other residues of the FabG dimer interfaces, may exhibit little affinity for the mammalian FAS KR domain.

### Reported inhibitors and potential lead compounds

Whilst current FabG inhibitors do not appear to have progressed to the stage of clinical trials, a number of lead molecules and inhibitors have been reported [4–8]. The most potent of these molecules in terms of inhibitory activity are a class of allosteric inhibitors reported by Cukier et al. (2013), with IC_50_ values ranging from 138 μM to 20 nM [8], that bind within the A/D and B/C dimer interfaces of FabG between helices α4 and α5 of each monomer (Fig 4B), and appear to distort the NADPH binding pocket sufficiently to inhibit binding of the co-factor. Whilst the allosteric inhibitors reported by Cukier et al. (2013) target the dimer interfaces of FabG and might be highly selective for bacterial FabG enzymes, neither the activity of these inhibitors against the mammalian FAS, or minimum inhibitory concentration (MIC) values against bacteria have been reported [8]. Furthermore, while the KR domain of the mammalian FAS complex does not possess the same dimer interfaces as bacterial FabG enzymes, the mammalian FAS does contain a region somewhat homologous to the A/D interface of *Yp*FabG and *E*. *coli* FabG, and thus potentially the binding site of these inhibitors, adjacent to the co-factor binding site. However, two of the four helices of this site or pseudo-interface are truncated in comparison to helices α4 and α5 (Fig 7B), and whether this structural difference is sufficient to prevent activity against the mammalian FAS is yet to be determined.

Similarly, Macrolactin B and Macrolactin S have been reported to inhibit *Staphylococcus aureus* FabG, with IC_50_ values of 100 μM and 130 μM respectively, and exhibit weak antibacterial activity against *E*. *coli*, *Bacillus subtilis* and *S*. *aureus*, with MIC_50_ values ranging from 64 to 128 μg mL^-1^. However, any structural data pertaining to the mechanism of inhibition of these Macrolactins, or the activity of these molecules against the mammalian FAS KR domain, if any, is yet to be reported [4].

The triclosan (a widely used FabI inhibitor) analogues hexachlorophene, bithionol, di-resorcinol sulphide, and bromochlorophene have all been shown to inhibit *P*. *falciparum* FabG, with IC_50_ values for these compounds ranging from 2.05 μM for hexachlorophene to 15.4 μM for bromochlorophene [7]. While no structural data detailing the binding of these molecules has been reported, hexachlorophene has been shown to inhibit *P*. *falciparum* FabG in a competitive mechanism in regards to NADPH, and a mixed inhibition mechanism in regards to the fatty acyl substrate [7]. Due to the highly similar structure of these molecules, it would seem likely that they would share a common mechanism of inhibition, and virtual screening or docking studies may provide an avenue for further investigation of these inhibitors or related molecules. A combination of molecular docking and *in vitro* analysis led to the discovery of another triclosan analogue (OAR27), able to inhibit *Schistosoma japonicum* FabG with an IC_50_ value of 51 μM. The compound (OAR27) displayed cytotoxicity in human liver carcinoma cells [6], indicating OAR27 may possess some activity against the KR domain of human FAS, yet this could possibly be reduced with optimisation using the structures of *Yp*FabG and other bacterial FabG enzymes as a basis for such drug design.

With the exception of the allosteric inhibitors reported by Cukier et al. (2013) [8], no structural data indicating the amino acids or functional groups required for the interactions between FabG and these inhibitors have been reported, thus the three-dimensional structure of FabG bound to these inhibitors, or rigorous virtual screening followed by site-directed mutagenesis to confirm any apparent interactions, would be necessary to allow a structure-based drug design approach. Once the binding site of these inhibitors and residues required for protein/inhibitor interaction are known, these compounds could be further altered to enhance the affinity for *Yp*FabG and bacterial FabGs, and reduce the affinity for the KR domain of mammalian FAS.

## Conclusion

Here we describe x-ray structures of FabG from *Yersinia pestis*. FabG is a highly conserved and ubiquitously expressed ketoacyl-ACP reductase of the bacterial FASII pathway, and a key component of bacterial lipogenesis. *Yp*FabG crystallised in two different crystal forms, one diffracting to 2.50 Å, and the other to 2.85 Å. The two crystal forms were highly similar, with an RMSD of 0.35 Å over 205 amino acids, yet displayed differences within two loop regions adjacent to the NADP(H) binding site. The structures of *Yp*FabG exhibit high structural homology with other SDRs (RMSD of 0.59–2.24 Å), including the ketoreductase domain of the mammalian FAS from *Homo sapiens* and *Sus scrofa*, despite sequence homologies from as little as 18%. However, further comparison of *Yp*FabG to the human and *S*. *scrofa* FAS enzymes also reveals structural differences surrounding the NADP(H) binding site and dimer interfaces, with the potential for such differences to be exploited during structure-based drug design of new antimicrobial agents to combat *Y*. *pestis* infections that exhibit poor affinity for mammalian homologues, thus limiting adverse host effects.
